# Effect of exogenous selenium supply on photosynthesis, Na^+^ accumulation and antioxidative capacity of maize (*Zea mays* L.) under salinity stress

**DOI:** 10.1038/srep42039

**Published:** 2017-02-07

**Authors:** Chaoqiang Jiang, Chaolong Zu, Dianjun Lu, Qingsong Zheng, Jia Shen, Huoyan Wang, Decheng Li

**Affiliations:** 1 Tobacco Research Institute/Maize Research Center, Anhui Academy of Agricultural Sciences, Hefei 230031, P. R. China; 2 State Key Laboratory of Soil and Sustainable Agriculture, Institute of Soil Science, Chinese Academy of Sciences, Nanjing 210008, P. R. China; 3 College of Natural Resources and Environmental Science, Key Laboratory of Marine Biology, Nanjing Agricultural University, Nanjing 210095, P. R. China

## Abstract

The mechanism of selenium-mediated salt tolerance has not been fully clarified. This study investigated the possible role of selenium (Se) in regulating maize salt tolerance. A pot experiment was conducted to investigate the role of Se (0, 1, 5 and 25 μM Na_2_SeO_3_) in photosynthesis, antioxidative capacity and ion homeostasis in maize under salinity. The results showed that Se (1 μM) relieved the salt-induced inhibitory effects on the plant growth and development of 15-day-old maize plants. Se application (1 μM) also increased the net photosynthetic rate and alleviated the damage to chloroplast ultrastructure induced by NaCl. The superoxide dismutase (SOD) and ascorbate peroxidase (APX) activities were increased, and *ZmMPK5, ZmMPK7* and *ZmCPK11* were markedly up-regulated in the roots of Se-treated plants, likely contributing to the improvement of antioxidant defence systems under salinity. Moreover, 1 μM Se increased K^+^ in the shoots while decreasing Na^+^ in the roots, indicating that Se up-regulates *ZmNHX1* in the roots, which may be involved in Na^+^ compartmentalisation under salinity. The findings from this single experiment require repetition together with measurement of reactive oxygen species (ROS), but nevertheless suggest that exogenous Se alleviates salt stress in maize via the improvement of photosynthetic capacity, the activities of antioxidant enzymes and the regulation of Na^+^ homeostasis.

Salinity stress is one of the major environmental threats that seriously limit plant growth and crop productivity. High salinity inhibits plant growth and development, principally due to osmotic stress and ionic toxicity^1^,^2^. The osmotic stress induced by salt stress can lead to a dramatic decrease in the stomatal aperture, which decreases the photosynthetic capacity^2^. Therefore, in many plants, growth inhibition is closely related to a decrease in photosynthesis under salt stress^3^,^4^,^5^,^6^. High salinity reduces the photosynthetic activity of plants and is associated with stomatal limitations, such as stomatal closure^2^,^7^, and/or non-stomatal limitations, including chlorophyll degradation^4^,^8^, chloroplast ultrastructure damage^9^,^10^, and the degradation of membrane and enzymatic proteins in the photosynthetic apparatus^3^,^11^. In addition, secondary stresses, such as oxidative stress, are often accompanied by osmotic stress and ionic toxicity, which are harmful to plant cells due to the accumulation of excessive reactive oxygen species (ROS)^2^,^12^. ROS can cause significant damage to membrane lipids, proteins, nucleic acids and photosynthetic pigments^13^. Therefore, the antioxidant capacity and photosynthetic capacity are highly important for normal plant growth and development under salt stress^2^,^6^.

Considerable efforts have been made to enhance plant salt tolerance, including the use of exogenous substances, such as nitric oxide^14^, polyamine^15^, brassinolide^16^, silicon^10^, melatonin^5^,^17^ and selenium (Se)^6^,^18^,^19^. Se is an essential micronutrient for animals and humans and appears to be a beneficial element for many plants^19^,^20^. The beneficial effect of Se at low concentrations has been well documented in potato^21^ and green algae^22^. More importantly, many studies have shown that Se can alleviate the detrimental effects on plants of diverse abiotic stresses, e.g., drought^23^, salt^6^,^24^, high temperature^25^ and heavy metals^26^. Most of these studies have suggested that Se might act as an antioxidant, alleviating oxidative stress induced by environmental stressors, thus improving abiotic stress tolerance in plants. In addition, Kong *et al*.^27^ demonstrated that Se application maintained chloroplast and mitochondria ultrastructures, thereby improving photosynthesis and enhancing salt tolerance in sorrel (*Rumex patientia* × *R. tianshanicus*) seedlings. Furthermore, Se application significantly reversed the negative effects of salinity on the photochemical efficiency of photosystem II (PSII) in tomato seedlings^6^. Based on previous observations, the positive effect of Se in plants is believed to be related to the regulation of antioxidant defence systems and photosynthesis. However, the information regarding the effects of exogenous Se supply on photosynthesis and on the antioxidant machinery of salt-stressed plants is limited and needs to be further explored.

Maize (*Zea mays* L.) is one of the most important food crops in the world and also provides raw material for industry^28^,^29^. Many studies have shown that maize production must double to meet the growing demands of human/animal consumption and biofuel production, particularly in developing countries^29^. Maize is known to be a moderately salt-sensitive plant, and its growth and yield are severely limited at high salinity^30^. Currently, more than 20% of the world’s irrigated land is threatened by salinity^31^, which will further limit the crop yield, including that of maize, in the future. In China, maize accounts for more than one-third of cereal production^29^; however, its growth and yield are severely limited at high salinity. Moreover, many major corn-producing areas of China are likely to be threatened by increasing soil salinisation resulting from climate change and water shortages^29^. Therefore, salinity has become one of the most serious threats to maize production^30^. Although several methods have been used to improve plant salt tolerance and to exploit the saline soil, new and more efficient ways to increase crop yield are important for sustainable agricultural development and food security, particularly in drought-prone and high-salinity areas.

The objectives of this study were to evaluate the effects of Se on plant growth, photosynthesis, the antioxidant system and ion homeostasis in NaCl-treated maize plants. Furthermore, physiological and molecular mechanisms for the roles of Se in improving plant salt tolerance were proposed. This systematic investigation provided more information to better understand the mechanisms of the Se-induced enhancement of plant salt tolerance.

## Results

### Plant growth and biomass of maize plant under selenium and NaCl

**Figure 1:**
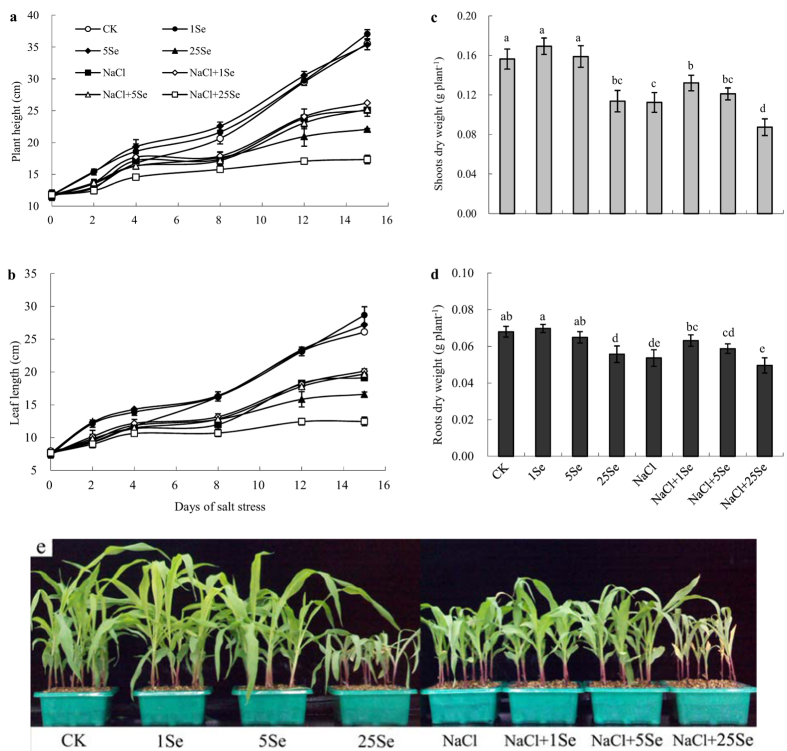
Effects of Se on plant height (**a**), leaf length (**b**), dry weight (**c**,**d**), and growth performance (**e**) of non-stressed or salt-stressed maize plants. The control plants (CK) were cultured in half-strength Hoagland’s solution. Different treatments were added with or without different concentrations of Se or NaCl. The abbreviations 1 Se, 5 Se and 25 Se indicate 1 μM Na_2_SeO_3_, 5 μM Na_2_SeO_3_ and 25 μM Na_2_SeO_3_, respectively (details are shown in the Materials and Methods). Data are presented as the means ± SDs (n = 18). Columns labelled with different letters between treatments represent significant differences (*p* < 0.05).

The shoot and root dry weights of maize were significantly affected by the application of NaCl and Se (*p* ≤ 0.001), the interaction of NaCl and Se application rate had no significant effects on the shoot and root dry weights (Table 1). As shown in Fig. 1, the application of 1 and 5 μM Se had no significant effect on the plant growth without salt treatment. However, when Se was applied at a concentration of 25 μM, the dry weights of the shoots and roots were reduced by 27% and 18%, respectively, compared with the control. In addition, NaCl reduced the dry weights of the shoots and roots by 28% and 21%, respectively, compared with the control (Fig. 1c and d). In salt-treated plants, the application of 1 μM Se significantly increased the dry weights of the shoots and roots by 17% and 18%, respectively, and Se-treated plants exhibited better growth (Fig. 1e). By contrast, 25 μM Se reduced plant height, leaf length and dry weight when plants were cultured with 100 mM NaCl.

### Photosynthetic pigments and gas exchange under selenium and NaCl

NaCl significantly affected chlorophyll and carotenoid contents in maize, while application rate of Se had significant effects on carotenoid content (Table 1). As shown in Table 2, NaCl treatment caused 24% and 14% decreases in chlorophyll and carotenoid content, respectively, compared with the control. In salt-treated plants, 1 μM Se-treated plants showed higher chlorophyll (13%) and carotenoid (7%) contents than the plants grown without Se application. However, the chlorophyll contents and the chlorophyll/carotenoid ratio were progressively decreased by 25 μM Se applications in both the salt-treated and non-treated plants.

NaCl and application of high Se markedly decreased the net photosynthetic rate (*P*_n_) in maize leaves, and the interaction of NaCl and Se significantly affected the *P*_n_ (Table 1). Under salt stress, Se application at concentrations of 1 μM increased *P*_n_ by 19% compared with non-Se-treated plants, whereas 25 μM Se progressively decreased *P*_n_ (Table 3). Similarly, the stomatal conductance (*G*_s_) and transpiration rate (*T*_r_) in the leaf were significantly reduced by salt stress, and the application of 1 μM Se alleviated these adverse effects under salt stress. In contrast, Se application had no significant effect on the intercellular CO_2_ concentration (*C*_i_), and even reduced the *C*_i_ in the maize leaf under salt stress (Table 3).

### Chloroplast ultrastructure in leaf in response to selenium and NaCl

**Figure 2:**
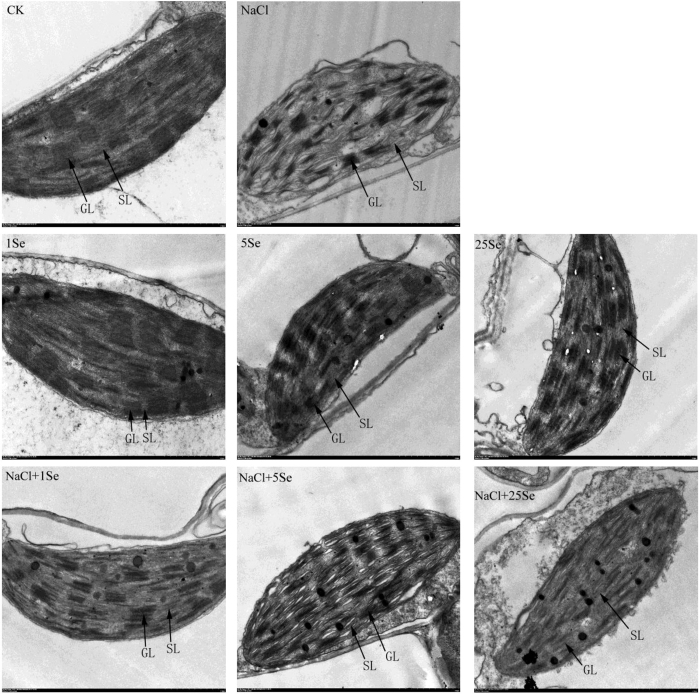
Effects of Se on the chloroplast ultrastructure of leaves of non-stressed or salt-stressed maize plants. The control plants (CK) were cultured in half-strength Hoagland’s solution. Different treatments were added with or without different concentrations of Se or NaCl. The abbreviations 1 Se, 5 Se and 25 Se indicate 1 μM Na_2_SeO_3_, 5 μM Na_2_SeO_3_ and 25 μM Na_2_SeO_3_, respectively (details are shown in the Materials and Methods).

As shown in Fig. 2, under salt stress, prominent damage was observed in the chloroplast ultrastructure of maize leaves. The chloroplasts were characterised by compressed grana lamellae, disrupted stroma lamella and distorted thylakoids. No significant differences in the ultrastructure of chloroplasts were observed between the control and low-concentration Se (1 and 5 μM) alone treatments, but 25 μM Se clearly damaged the chloroplast ultrastructure. However, 1 μM Se application alleviated the structural damage to the chloroplasts induced by NaCl, resulting in a more integrated internal lamella, a thicker grana lamellae, and a more regular shape of the thylakoids in the leaf than in the plants treated with NaCl alone.

### Cell membrane damage and antioxidant enzymes activity in response to selenium and NaCl

**Figure 3:**
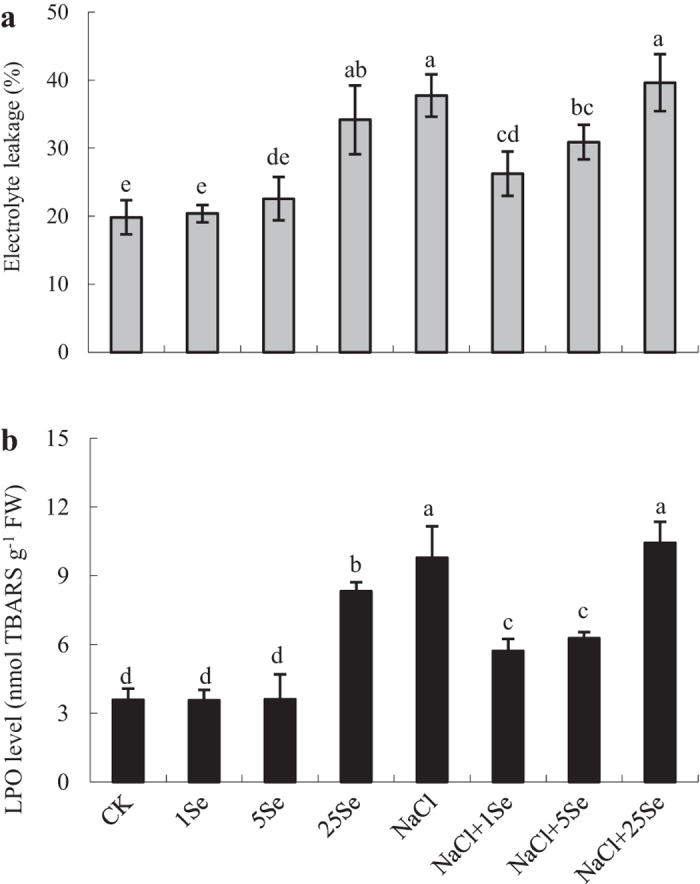
Effects of Se on electrolyte leakage (**a**) and LPO levels (**b**) in the leaves of non-stressed or salt-stressed maize plants. The control plants (CK) were cultured in half-strength Hoagland’s solution. Different treatments were added with or without different concentrations of Se or NaCl. The abbreviations 1 Se, 5 Se and 25 Se indicate 1 μM Na_2_SeO_3_, 5 μM Na_2_SeO_3_ and 25 μM Na_2_SeO_3_, respectively (details are shown in the Materials and Methods). Data are presented as the means ± SDs (n = 3). Columns labelled with different letters between treatments represent significant differences (*p* < 0.05).

Salt-treated plants had significantly higher electrolyte leakage (1.9-fold) and lipid peroxidation (LPO) levels (2.7-fold) than the control plants (Fig. 3 and Table 1). No significant differences in electrolyte leakage were observed among plants grown with 1 and 5 μM Se and without Se application under non-salt stress conditions, but electrolyte leakage was significantly increased by 100% in plants treated with 25 μM Se. Under salt stress, the application of 1 and 5 μM Se decreased electrolyte leakage by 30% and 18%, respectively, compared with plants without Se application (Fig. 3a). Similarly, under salt stress, the LPO levels in 1 and 5 μM Se-treated plants decreased by 42% and 36%, respectively, compared with non-Se-treated plants.

**Figure 4:**
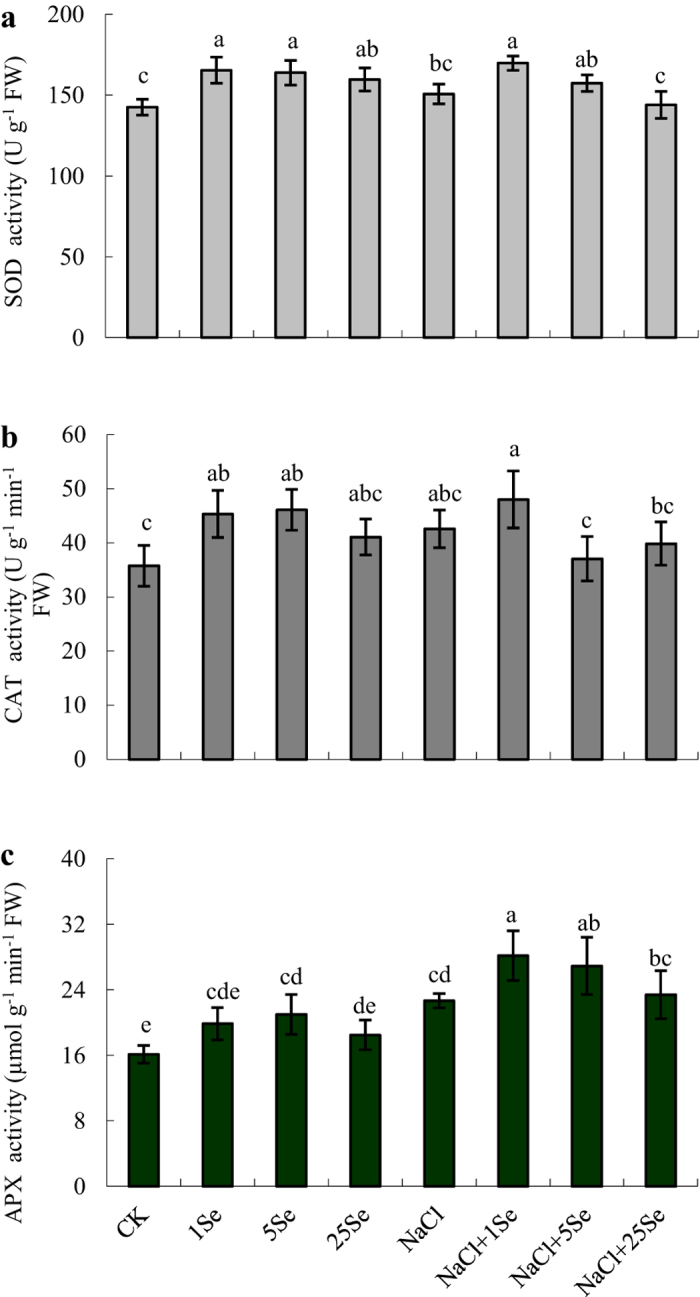
Effects of Se on SOD (**a**), CAT (**b**) and APX (**c**) activity levels in the leaves of non-stressed or salt-stressed maize plants. The control plants (CK) were cultured in half-strength Hoagland’s solution. Different treatments were added with or without different concentrations of Se or NaCl. The abbreviations 1 Se, 5 Se and 25 Se indicate 1 μM Na_2_SeO_3_, 5 μM Na_2_SeO_3_ and 25 μM Na_2_SeO_3_, respectively (details are shown in the Materials and Methods). Data are presented as the means ± SDs (n = 3). Columns labelled with different letters between treatments represent significant differences (*p* < 0.05).

The application of Se significantly affected superoxide dismutase (SOD), catalase (CAT) and ascorbate peroxidase (APX) in maize, but NaCl only significantly affected the APX (Table 1). As showed in Fig. 4, Se activated the SOD enzyme in plants grown with or without NaCl, particularly at 1 and 5 μM Se concentrations. Under salt stress, SOD activity was increased by 13% with the application of 1 μM Se, but a higher Se concentration (25 μM) decreased its activity (Fig. 4a). Similarly, NaCl increased CAT activity by 19%, and 1 μM Se increased its activity by 27% in non-salt-treated plants. However, an obvious increase in APX activity was detected in Se-treated plants grown with or without NaCl. More importantly, under salt stress, APX activity increased by 24% and 19% in 1 and 5 μM Se-treated plants, respectively, compared with non-Se-treated plants (Fig. 4c).

### *ZmMPK5, ZmMPK7* and *ZmCPK11* expression in response to selenium and NaCl

**Figure 5:**
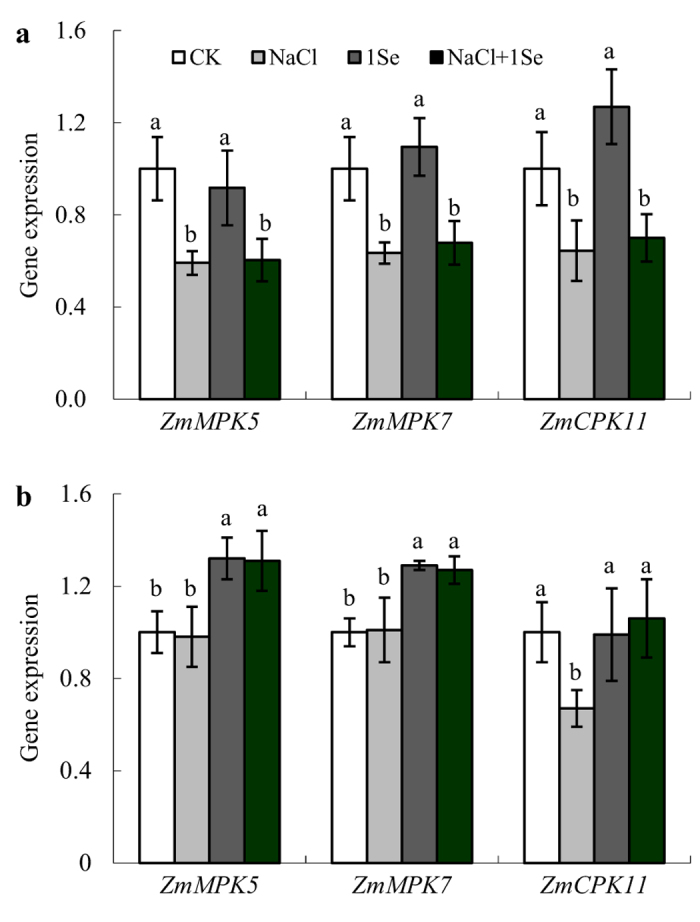
Real-time quantitative PCR analysis of *ZmMPK5, ZmMPK7* and *ZmCPK11* mRNA accumulation in the roots of maize plants treated with NaCl (0 and 100 mM) and Se (0 and 1 μM) for 2 h (**a**) or 24 h (**b**). CK: half-strength Hoagland’s solution; NaCl: half-strength Hoagland’s solution + 100 mM NaCl; 1 Se: half-strength Hoagland’s solution + 1 μM Na_2_SeO_3_; NaCl + 1 Se: half-strength Hoagland’s solution + 100 mM NaCl + 1 μM Na_2_SeO_3_. Data are presented as the means ± SDs (n = 3). For each gene expression, columns labelled with different letters between treatments represent significant differences (*p* < 0.05).

We further examined the *ZmMPK5, ZmMPK7* and *ZmCPK11* gene expression levels in the roots at 2 and 24 h after treatment (Fig. 5). Under salt stress, *ZmMPK5, ZmMPK7* and *ZmCPK11* were significantly down-regulated in the roots at 2 h (Fig. 5 and Table 1, *p* ≤ 0.001); *ZmCPK11* was also down-regulated at 24 h. Similarly, the application of 1 μM Se significantly down-regulated all of these genes at 2 h under salt stress (Fig. 5a). However, Se application caused a clear increase in *ZmMPK5* and *ZmMPK7* up-regulation in the roots at 24 h. Under salt stress, the relative expression levels of *ZmMPK5* and *ZmMPK7* in roots treated with 1 μM Se were approximately 1.3-fold those found in non-Se-treated plants, and *ZmCPK11* expression also increased by 58% (Fig. 5b).

### K^+^, Na^+,^ Cl^−^ and Se contents and *ZmNHX1* expression under selenium and NaCl

NaCl significantly affected Na^+^, Cl^−^ and K^+^ contents in both shoot and root of maize plant (*p* ≤ 0.001), and Se application significantly affected K^+^ contents in both shoot and root, and Na^+^ contents in root (*p* ≤ 0.05) (Table 1). The interaction of NaCl and Se only significantly affected K^+^ content in shoot and Na^+^ contents in root (*p* ≤ 0.05) (Table 1). As shown in Table 4, no significant differences in K^+^ contents were detected in the shoots and roots of 1 and 5 μM Se-treated plants compared with control plants, whereas 25 μM Se reduced the K^+^ content. Under salt stress, the K^+^ contents in the shoots and roots were decreased by 34% and 61%, respectively. However, clear increases in K^+^ content were observed in the shoots of 1 and 5 μM Se-treated plants grown with NaCl. By contrast, salt-treated plants exhibited Na^+^ content at least 6.4-fold higher than in control plants, whereas the application of 1 and 5 μM Se decreased Na^+^ content in the roots by approximately 15% in plants grown with NaCl. Thus, the K^+^/Na^+^ ratios, which were high in the control plants, decreased greatly under salt stress. The K^+^/Na^+^ ratios were not significantly affected by Se application. In addition, NaCl markedly increased Cl^−^ contents in the shoots and roots, but Se application had no significant effect on Cl^−^ contents in maize.

**Figure 6:**
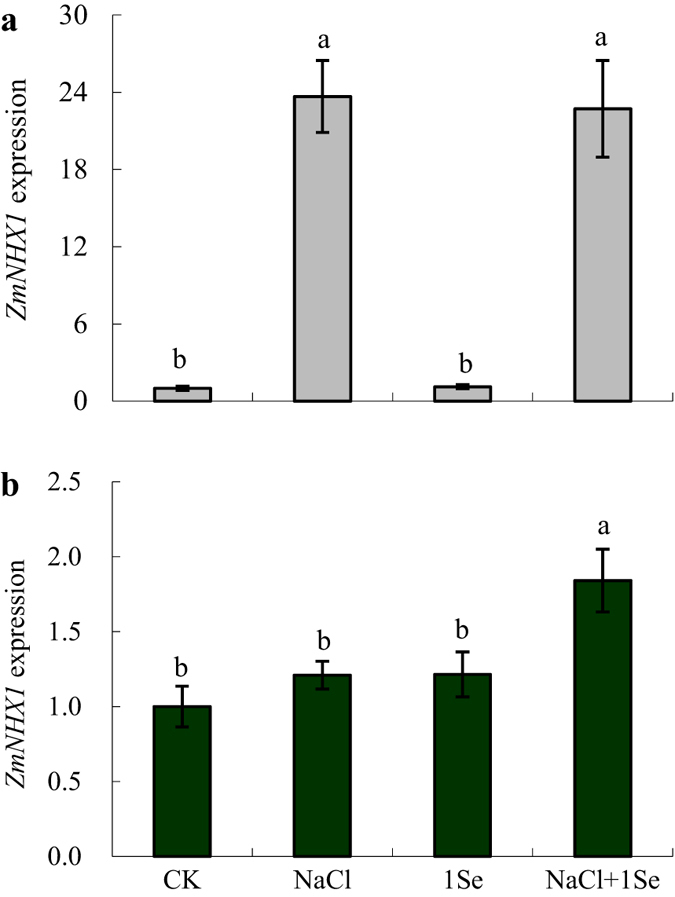
Real-time quantitative PCR analysis of *ZmNHX1* mRNA accumulation in the roots of maize plants treated with NaCl (0 and 100 mM) and Se (0 and 1 μM) for 2 h (**a**) or 24 h (**b**). CK: half-strength Hoagland’s solution; NaCl: half-strength Hoagland’s solution + 100 mM NaCl; 1 Se: half-strength Hoagland’s solution + 1 μM Na_2_SeO_3_; NaCl + 1 Se: half-strength Hoagland’s solution + 100 mM NaCl + 1 μM Na_2_SeO_3_. Data are presented as the means ± SDs (n = 3). Columns labelled with different letters between treatments represent significant differences (*p* < 0.05).

To investigate whether Se is involved in Na^+^ and K^+^ homeostasis under salt stress, the *ZmNHX1* expression in the roots was examined. As shown in Fig. 6, NaCl significantly induced *ZmNHX1* expression in the roots at 2 h but had no significant effect on the expression of this gene after 24 h of treatment. However, *ZmNHX1* expression in 1 μM Se-treated maize roots was significantly increased at 24 h after salt stress (Table 1, *p* = 0.002).

**Figure 7:**
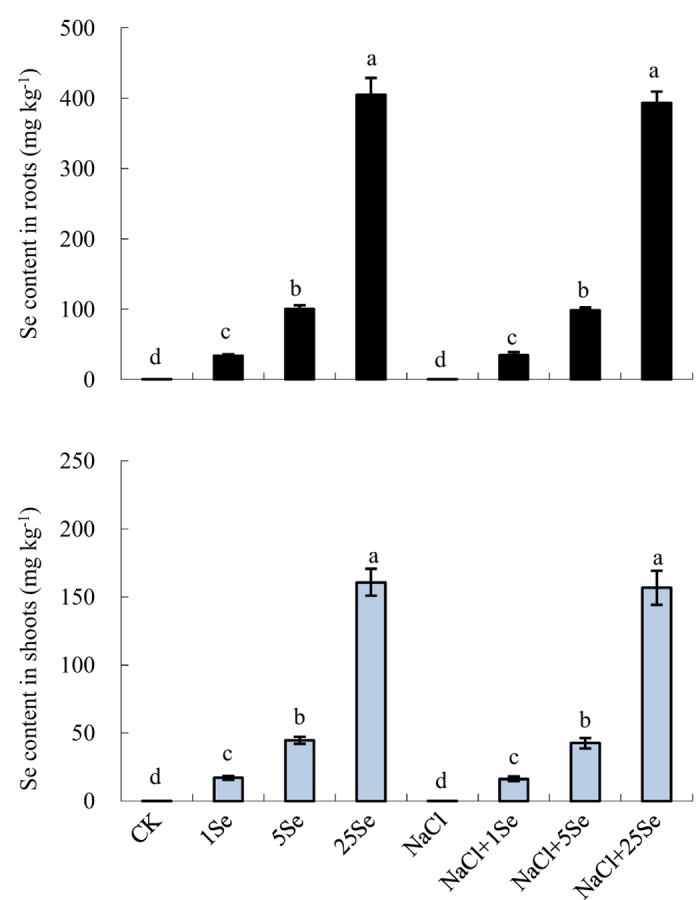
Effects of exogenous Se on Se content in the shoots and roots of non-stressed or salt-stressed maize plants. The control plants (CK) were cultured in half-strength Hoagland’s solution. Different treatments were added with or without different concentrations of Se or NaCl. The abbreviations 1 Se, 5 Se and 25 Se indicate 1 μM Na_2_SeO_3_, 5 μM Na_2_SeO_3_ and 25 μM Na_2_SeO_3_, respectively (details are shown in the Materials and Methods). Data are presented as the means ± SDs (n = 3). Columns labelled with different letters between treatments represent significant differences (*p* < 0.05).

The Se contents in the shoots and roots increased exponentially with increasing Se application rate (Fig. 7 and Table 1, *p* ≤ 0.001). For example, under non-salt treatments, Se application at a concentration of 5 μM caused the Se content in the shoots to increase from 17.11 mg kg^−1^ to 44.74 mg kg^−1^ relative to the 1 μM Se treatment. Moreover, Se content in the shoots increased to 160.79 mg kg^−1^ after treatment with 25 μM Se. However, there were no significant effects of NaCl on the Se content.

## Discussion

Salt stress has been extensively shown to severely reduce plant growth and limit crop production^2^. Maize is considered a moderately salt-sensitive crop; therefore, its growth and yield are severely reduced by salinity^30^. In this study, plant height, leaf length and dry weight were significantly reduced in maize plants grown with 100 mM NaCl for 15 days (Fig. 1). However, the growth inhibition under salt stress was alleviated by the application of Se (1 μM) (Fig. 1). Se is considered to be beneficial for plant growth and development, particularly in hyperaccumulators^32^. Previous studies have shown that Se improves plant salt tolerance^6^,^18^,^19^,^24^. Hawrylak-Nowak^18^ found that Se application (5 and 10 μM) significantly increased the plant dry weight of NaCl-treated cucumber seedlings, and Diao *et al*.^6^ also observed that exogenous Se significantly increased the dry weight of tomato seedlings under salinity. In this current study, the application of 1 μM Se improved the tolerance of maize to salt stress, showing that Se increases the plant height, leaf length and biomass of maize under salinity. Therefore, the results of this study further confirmed the beneficial effects of Se in plants subjected to stress conditions. By contrast, the application of 25 μM Se resulted in significant decreases in plant height, leaf length and biomass accumulation. Similar results were observed by Lehotai *et al*.^33^, who showed that a high Se concentration (40 μM) significantly inhibited the root growth of *Arabidopsis thaliana*, and Hawrylak-Nowak^18^ observed that high levels of Se (20 μM) had no significant effect on the plant growth of cucumber seedlings. Thus, Se has dual effects on maize plants: it stimulates plant growth at low concentrations but is toxic to plant growth and development at high concentrations.

Similar to plant growth, photosynthesis is one of the primary processes affected by salinity^2^. This study observed that the photosynthesis in maize leaves was reduced under salt stress. However, the application of low concentrations of Se (1 μM) significantly increased the net photosynthetic rate in maize plants grown under salt stress (Table 3). The improvement of photosynthesis in response to Se has also been observed in tomato under salt stress^6^ and in sorghum under high-temperature stress^25^. In addition, 1 μM Se-treated plants showed higher chlorophyll content than non-Se-treated plants under salt stress (Table 2), and the damage to chloroplasts induced by NaCl was alleviated by Se application (Fig. 2). Similar effects of Se on chlorophyll content and chloroplast ultrastructure have been detected in sorrel (*Rumex patientia* × *R. tianshanicus*)^27^, cucumber^18^ and tomato^6^ under salt stress. Furthermore, Se accelerated chlorophyll biosynthesis by facilitating respiration and electron transport in the respiratory chain^34^. Therefore, restoration of the photosynthetic capacity in salt-treated maize plants by Se application may be related to the increases in chlorophyll content and the preservation of chloroplast ultrastructure. However, this study observed that a high Se concentration (25 μM) aggravated the damage to the photosynthetic system in maize plants, resulting in further decreases in net photosynthetic rate, stomatal conductance, transpiration rate and chlorophyll content, as well as more drastic damage to the chloroplast ultrastructure. Therefore, low concentrations of Se (1 μM) might improve photosynthesis in maize plants and result in better growth under salinity.

It is well established that salt stress causes lipid peroxidation and might result in increases in membrane permeability and ion leakage^14^. In this study, salt stress resulted in severe damage to the maize plants, and corresponding increases in electrolyte leakage and LPO levels were observed in maize leaves under salt stress. However, 1 μM Se significantly decreased the electrolyte leakage and LPO levels in salt-treated plants (Fig. 3). Electrolyte leakage is commonly believed to be an important index of cell membrane permeability. Lower lipid peroxidation resulting from elevated activities of antioxidants under salt stress was also reported in silicon-treated cucumber under salt stress^35^. Recently, Diao *et al*.^6^ found that Se application significantly enhanced the antioxidant defence system in the chloroplasts of tomato seedlings under salt stress, which could be responsible for the improvement in photosynthesis. These results further confirm that Se plays important roles in the maintenance of cell membrane structure and cell integrity under salt stress. The current results on the chloroplast ultrastructure in Se-treated leave also confirm this observation (Fig. 2).

Salt stress causes excess ROS and results in oxidative stress primarily due to the overproduction of active oxygen^2^,^12^. Excess ROS formation induces lipid peroxidation, severely impairs proteins and DNA, thus inhibiting signal transduction, and could interfere with normal cellular function, even resulting in cell death^36^. Previous studies have demonstrated that exogenous Se can increase the activity of antioxidant enzymes and the tolerance of plants subjected to various stresses^6^,^24^,^27^. Under salt stress, clear increases in SOD and APX activity levels were observed in Se-treated plants compared with non-Se-treated plants (Fig. 4). These observations were consistent with the findings in sorrel^27^, rapeseed^24^ and tomato seedlings^6^ under salt stress. The activity levels of SOD, CAT and APX are crucial for scavenging ROS in plants. More importantly, the activity levels and balance of these enzymes are thought to be integral in converting the superoxide radical and hydrogen peroxide and in protecting plants from environmental stress^36^. SOD is believed to serve as the first line of antioxidant defence against ROS by catalysing superoxide to H_2_O_2_ and molecular oxygen^37^. In addition, APX might affect the fine modulation of ROS in signalling, whereas CAT is predominantly responsible for scavenging excess ROS^19^,^36^. In this study, under salt stress, increases in the SOD and APX activity levels coincided with a significant reduction in the damage to cell membranes in Se-treated plants compared with non-Se-treated plants (Figs 3 and 4). Although ROS should be further examined, the application of Se to salt-treated plants at a concentration of 1 μM induced the antioxidant defence systems to improve plant growth and development.

To further investigate the mechanisms by which Se induces antioxidant defence, the expression of related genes was examined. *ZmMPK5* and *ZmMPK7* and *ZmCPK11* were significantly up-regulated in the roots by 1 μM Se application with or without salt stress after 24 h (Fig. 5b). Previous studies have indicated that *ZmMPK5* is activated by H_2_O_2_, which up-regulated the antioxidant defence systems in the leaves of maize plants^38^. Recently, Ding *et al*.^39^ found that transient expression of *ZmCPK11* can increase the expression and activity levels of antioxidant enzymes (SOD and APX) in maize. Moreover, lower H_2_O_2_ accumulation and clear alleviation of ROS injuries were observed in *ZmMPK7-*overexpressing tobacco plants under osmotic stress^40^. In addition, Shi *et al*.^41^ observed that *GhMPK7* is involved in SA-regulated broad-spectrum resistance to pathogen infection, thereby regulating plant growth and development. Taken together, our results show that Se induced the expression of antioxidant defence genes, which in turn led to an increase in the antioxidant defence systems of maize plants under salt stress. However, the involvement of Se in the antioxidant defence genes and how these genes regulate antioxidant defence systems in Se-treated plants under salt stress need to be further analysed in the future.

Plant salt tolerance also depends on the maintenance of K^+^ and Na^+^ homeostasis^1^,^2^. Salt stress often causes a significant increase in Na^+^ accumulation and a considerable decrease in K^+^. thus markedly decreasing the K^+^/Na^+^ ratio^1^,^18^. A high level of intracellular K^+^ is crucial for the activity levels of many cytosolic enzymes, the maintenance of the membrane potential and osmoticum synthesis^1^. In this study, under salt stress, the application of 1 and 5 μM Se caused substantial increases in the K^+^ content in the shoots compared with non-Se-treated plants. These results are consistent with the findings of Kong *et al*.^27^, which showed that Se application increased K^+^ accumulation in salt-stressed sorrel plants. However, Hawrylak-Nowak^18^ demonstrated that Se did not significantly affect K^+^ accumulation in NaCl-stressed cucumber seedlings. In addition, this study showed that the K^+^/Na^+^ ratio was not significantly affected by the application of Se under salt stress. To further understand the effect of Se on ion accumulation in maize plants under salt stress, we analysed the expression of *ZmNHX1*, which is involved in Na^+^ compartmentalisation in root vacuoles^42^,^43^. *ZmNHX1* expression in the roots was up-regulated by Se at 24 h after salinity treatment (Fig. 6). Previous studies proposed that NHX functions in Na^+^ compartmentalisation^1^, and NHX overexpression in transgenic plants, such as tomato^44^, *Brassica napus*^45^ and poplar^4^, has been observed to enhance plant salt tolerance. These results suggest that Se up-regulates *ZmNHX1* in the roots, which may participate in Na^+^ compartmentalisation under salt stress. However, how Se regulates K^+^ uptake and accumulation under salt stress is currently unknown, which prevents us from drawing further conclusions on ion homeostasis based on Se application.

**Figure 8:**
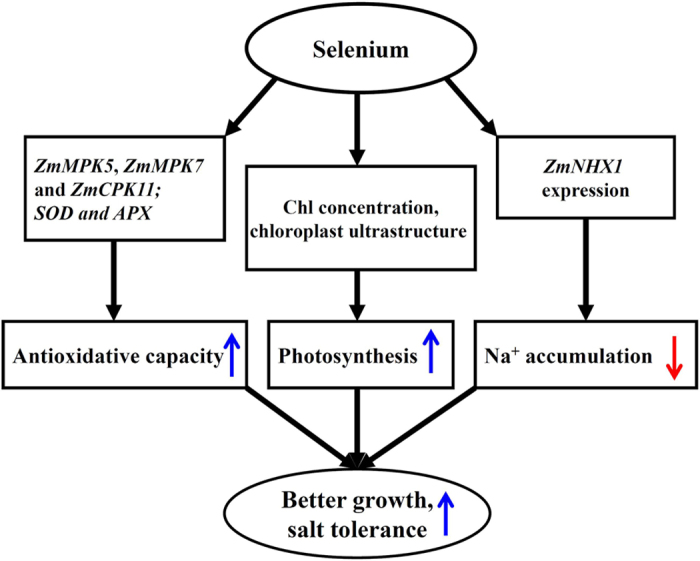
Schematic representation of the positive role of Se on salt tolerance of maize. In the present study, a model was developed to show that photosynthesis, antioxidant defence systems and Na^+^ accumulation were regulated by Se in maize under salt stress. The blue arrows (↑) and the red arrows (↓) represent the positive and passive role of Se, respectively.

In conclusion, the findings from this single experiment require repetition together with measurement of ROS, but nevertheless indicate that the application of low levels of Se (1 μM) alleviated the inhibitory effect of high salinity via three mechanisms (Fig. 8): (a) the improvement in photosynthetic capacity by the decrease in chlorophyll degradation and the preservation of chloroplast ultrastructure, (b) the activation of the antioxidant defence system to alleviate ROS damage, and (c) the amelioration of ion homeostasis in maize plants under salt stress, particularly Na^+^ compartmentalisation. Thus, this study provides evidence that low Se application can enhance plant salt tolerance, and our results increase the understanding of the precise role of Se in the response of plants to salinity.

## Materials and Methods

### Experimental layout

Maize seeds (*Zea mays* L. cv. Nongda 108) were surface-sterilised with 0.1% (v/v) HgCl_2_ for 5 min, washed repeatedly with distilled water, and germinated on wet filter paper in glass petri dishes for 2 days. After germination, seedlings were selected, transferred to plastic pots (14.5 cm × 14.5 cm × 11 cm, 9 plants per pot) filled with vermiculite and watered with half-strength Hoagland solution. The seedlings were placed in a cultivation chamber (GMC-250, Shanghai Yiheng Technical Co., Ltd., China) under controlled environmental conditions with temperatures of 28 °C (day) and 22 °C (night), relative humidity of 65%, and a photoperiod of 14 h/10 h at a photosynthetic photon-flux density of 200 μmol m^−2^ s^−1^. After the plants had 2 fully expanded leaves, uniform seedlings were subjected to different treatments by adding NaCl (0 or 100 mM) and Se (0, 1, 5 or 25 μM) dissolved in half-strength Hoagland solution (Table 5). The concentrations of Se were selected according to the effect of different Se concentrations (0.1, 0.5, 1, 5, and 10 μM Na_2_SeO_3_) on the growth of maize plants in a preliminary study, which showed that 1 μM Se application significant promoted shoot growth of maize after 8 days of treatment (data no showed). In this study, selenite (Na_2_SeO_3_) was used as the selenium source because it is more efficient than selenate in alleviation of salt stress^46^. The experiment included 8 treatments with 3 replicates (36 plants in total of each treatment). Each replicate included 2 pots of 12 plants (6 uniform plants were selected from the 9 plants in each pot). The nutrient solutions containing Se and NaCl were changed every two days. After 15 days of treatments, the plant biomass was determined.

### Plant growth measurements and chemical analyses

Plant height and leaf length were measured with a ruler at 0, 2, 4, 8, 12 and 15 days after the start of treatment. After 15 days of the treatments, the plants were divided into shoots and roots, and dry weights (DW) were obtained after oven-drying at 65 °C for 24 h or until constant weight. The oven-dried shoots and roots were finely ground and passed through a 1-mm-diameter sieve. K^+^ and Na^+^ contents were determined by flame emission spectrophotometry (FP 640, Shanghai Xinyi Instrument Co., Ltd., China) after the samples were extracted with 1 M HNO_3_ according to the method described by Storey^47^. The Cl^−^ contents were determined according to the method of Jiang *et al*.^48^. The shoots and roots samples were digested by the previously reported method^49^, and the total Se concentration in the solution was analysed using inductively coupled plasma mass spectrometry (X Series ICP–MS (Thermo Electron Corporation, United States). Eighteen plants of each treatment were measured for plant height, leaf length and dry weight. And these eighteen plants divided into 3 uniform groups (6 plants in each group) of each treatment were measured for ion concentrations (K^+^, Na^+^, Cl^−^ and Se).

### Photosynthetic pigment concentrations and gas exchange measurements

On day 15 of the salt treatment, the chlorophyll and carotenoid concentrations were determined using an ultraviolet spectrophotometer (UV765, Shanghai Precision and Scientific Instrument Co., Ltd., China). New fully expanded leaf samples were homogenised with 95% (v/v) ethanol containing 2.5 mM sodium phosphate buffer (pH 7.8), followed by centrifugation. The absorbance was measured at 665 and 649 nm for chlorophyll a and chlorophyll b, respectively, using an ultraviolet spectrophotometer (UV765, Shanghai Precision and Scientific Instrument Co., Ltd., China). Chlorophyll and carotenoid concentrations were calculated using the equations of Arnon^50^. The gas exchange of new fully expanded leaves was determined using a portable photosynthesis open system LI-6400XT (Li-Cor Inc., USA) from 9:00 to 12:00, according to the method of Jiang *et al*.^4^. During the measurements, the cuvette temperature was maintained at 22 ± 1 °C and a relative humidity of 50 ± 5%, with a photosynthetic photon flux intensity (PPFD) of 1000 μmol m^−2^ s^−1^. Data were recorded after equilibration to a steady state. Twelve plants divided into 3 uniform groups (4 plants in each group) of each treatment were used for chlorophyll and carotenoid concentrations, and four uniform plants of each treatment were used for gas exchange measurements.

### Transmission electron microscopy

Leaf segments of newly expanded leaves were cut from uniform treated plants of each treatment with a knife blade and fixed in 2.5% (w/v) glutaraldehyde in 100 mM phosphate buffer (pH 7.2). After washing in 100 mM phosphate buffer, the samples were post-fixed in 2% (w/v) osmium tetroxide, then dehydrated in an acetone series and embedded in epoxy resin. Leaf sections (70 nm) were cut using a Power Tome-XL ultra-microtome and stained with 2% (w/v) uranyl acetate followed by 5% lead citrate. Samples were observed using a transmission electron microscope (TEM) (HT-7700, Hitachi, Japan) at 80 kV.

### Electrolyte leakage and LPO levels measurements

The electrolyte leakage of newly expanded leaves was determined using a conductivity metre (CT3031, Hangzhou Sinomeasure Automation Technology Co., Ltd., China) according to the method of Zhang *et al*.^14^. LPO of the newly expanded leaves was estimated by measuring the concentration of thiobarbituric acid reactive substances (TBARS), as described by Zhu *et al*.^35^. The lipid peroxides was expressed as nmol TBARS g^−1^ FW, using an extinction coefficient of 155 mM^−1^ cm^−1^. Twelve plants divided into 3 uniform groups (4 plants in each group) of each treatment were used in electrolyte leakage and LPO levels measurements.

### Extraction and assays of antioxidant enzymes

Fresh segments (0.5 g) of newly expanded leaves were ground into a fine powder in liquid N_2_ with a mortar and pestle. The powder was homogenised in 50 mM phosphate buffer (pH 7.0). The homogenate was centrifuged at 4 °C for 20 min at 15,000 × g, and the supernatant was used for subsequent enzyme assays^51^. SOD activity was determined according to Kong *et al*.^27^. One unit of SOD activity was defined as the amount of enzyme corresponding to 50% inhibition of NBT reduction. CAT activity was determined from the consumption of H_2_O_2_ at 240 nm for 3 min^51^. APX activity was determined by assessing the consumption of ascorbate at 240 nm for 60 s^35^. Twelve plants divided into 3 uniform groups (4 plants in each group) of each treatment were measured for antioxidant enzymes activities.

### Total RNA extraction and gene expression analysis

Total RNA samples were prepared from plant roots of 3 uniform groups (2 plants in each group) in each treatment using TRIzol (Invitrogen Inc., CA, USA) according to the manufacturer’s instructions. First-strand cDNA was synthesised from the total RNA (1 μg) by reverse transcription using oligo-dT primers and Superscript II reverse transcriptase (Invitrogen), according to the manufacturer’s instructions. Quantitative PCR was performed on a Bio-Rad CFX96 real-time PCR system using SYBR Green master mix (SYBR Premix Ex Taq^TM^ II; TaKaRa Bio; http://www.takara-bio.com), according to the manufacturer’s instructions (TaKaRa Biotechnology). Actin was used as an internal standard for mRNA expression. The primers used for qRT-PCR are shown in Table 6.

### Statistical analysis

All treatments and measurements were conducted at least in triplicate. Data were analysed using the SPSS 19.0 (SPSS, Inc., Chicago, USA) and are presented as the means ± SDs. Two-way ANOVA was used to assess the effects of NaCl and application rates of Se on maize dry weight, photosynthesis, ion accumulation, antioxidative capacity and related gene expression. Differences among treatments were compared by Fisher’s least significance difference (LSD) test at the 5% level.

## Additional Information

**How to cite this article**: Jiang, C. *et al*. Effect of exogenous selenium supply on photosynthesis, Na^+^ accumulation and antioxidative capacity of maize (*Zea mays* L.) under salinity stress. *Sci. Rep.* **7**, 42039; doi: 10.1038/srep42039 (2017).

**Publisher's note:** Springer Nature remains neutral with regard to jurisdictional claims in published maps and institutional affiliations.

## Figures and Tables

**Table 1:** Results of two-way ANOVA (*p* values) from the effects of NaCl, selenium (Se) and NaCl × Se interaction, on plant dry weight (Shoot DW: shoot dry weight; Root DW: root dry weight), photosynthetic pigment concentrations (chlorophyll, carotenoid), photosynthetic parameters (*P*_n_: net photosynthesis; *G*_s_: stomatal conductance; *C*_i_: intercellular CO_2_ concentration; *T*_r_: transpiration rate), electrolyte leakage, lipid peroxidation (LPO), antioxidant enzyme activity (SOD, CAT and APX), gene expression levels (*MPK5, MPK7, CPK11* and *NHX1*) and ion concentrations (K^+^, Na^+^, Cl^−^, and Se).

Effect	DF	Shoot DW	Root DW	Chlorophyll	Carotenoid	Chlorophyll/carotenoid	*P*_n_	*G*_s_	*C*_i_	*T*_r_	Electrolyte leakage	LPO
NaCl	1	<0.001	<0.001	<0.001	<0.001	0.022	<0.001	<0.001	<0.001	<0.001	<0.001	<0.001
Se	3	<0.001	<0.001	<0.001	0.816	0.218	<0.001	<0.001	0.060	<0.001	<0.001	<0.001
NaCl × Se	3	0.319	0.128	0.120	0.747	0.703	0.003	0.208	0.083	0.404	0.017	0.001
					2 h				24 h			
		SOD	CAT	APX	*MPK5*	*MPK 7*	*CPK11*	*NHX1*	*MPK5*	*MPK 7*	*CPK11*	*NHX1*
NaCl	1	0.380	0.913	<0.001	0.001	<0.001	<0.001	<0.001	0.786	0.856	0.172	0.002
Se	3	<0.001	0.028	0.008	0.620	0.290	0.082	0.767	0.001	<0.001	0.059	0.002
NaCl × Se	3	0.027	0.023	0.657	0.506	0.692	0.226	0.703	0.974	0.724	0.047	0.050
		Shoot					Root					
		K^+^	Na^+^	K^+^/Na^+^	Cl^−^	Se	K^+^	Na^+^	K^+^/Na^+^	Cl^−^	Se	
NaCl	1	<0.001	<0.001	<0.001	<0.001	0.472	<0.001	<0.001	<0.001	<0.001	0.474	
Se	3	0.002	0.816	0.447	0.621	<0.001	0.024	0.008	0.516	0.659	<0.001	
NaCl × Se	3	0.015	0.747	0.512	0.539	0.940	0.279	0.004	0.563	0.387	0.727	

For shoot DW and root DW, n = 18; for chlorophyll and carotenoid concentrations, n = 3; for *P*_n_, *G*_s_, *C*_i_ and *T*_r_, n = 4; for electrolyte leakage, LPO, SOD, CAT and APX, n = 3; for *MPK5, MPK7, CPK11* and *NHX1*, n = 3; for K^+^, Na^+^, Cl^−,^ and Se concentrations, n = 3.

**Table 2:** Effects of Se on chlorophyll and carotenoid concentrations in non-stressed or salt-stressed maize seedlings.

Treatment	Chlorophyll		Carotenoid		Chlorophyll/carotenoid
	Mean (mg g^−1^ FW)	Percentage of CK (%)	Mean (mg g^−1^ FW)	Percentage of CK (%)	
CK	1.84 ± 0.12ab		0.28 ± 0.01ab		6.61 ± 0.42a
1Se	1.94 ± 0.12a	105.42	0.29 ± 0.01a	103.54	6.72 ± 0.12a
5Se	1.76 ± 0.11b	95.62	0.28 ± 0.01a	101.61	6.24 ± 0.59ab
25Se	1.49 ± 0.06cd	81.24	0.25 ± 0.03bc	88.20	6.17 ± 0.99ab
NaCl	1.40 ± 0.09de	76.37	0.24 ± 0.02c	86.27	5.85 ± 0.09ab
NaCl + 1Se	1.58 ± 0.05c	85.99	0.26 ± 0.02abc	91.99	6.20 ± 0.53ab
NaCl + 5Se	1.55 ± 0.07cd	84.31	0.25 ± 0.02abc	91.60	6.11 ± 0.49ab
NaCl + 25Se	1.30 ± 0.10e	70.92	0.24 ± 0.02c	85.79	5.46 ± 0.10b

The control plants (CK) were cultured in half-strength Hoagland’s solution. Different treatments were added with or without different concentrations of Se or NaCl. The abbreviations 1 Se, 5 Se and 25 Se indicate 1 μM Na_2_SeO_3_, 5 μM Na_2_SeO_3_ and 25 μM Na_2_SeO_3_, respectively (details are shown in the Materials and Methods). Data are presented as the means ± SDs (n = 3). Different letters within a column represent significant differences (*p* < 0.05).

**Table 3:** Effects of Se on photosynthesis in non-stressed or salt-stressed maize seedlings.

Treatments	*P*_n_ (μmol CO_2_ m^−2^ s^−1^)	*G*_s_ (mol H_2_O m^−2^ s^−1^)	*C*_i_ (μmol CO_2_ mol^−1^)	T_r_ (mmol H_2_O m^−2^ s^−1^)
CK	10.69 ± 0.61a	0.11 ± 0.02a	267.87 ± 26.74ab	1.31 ± 0.19a
1Se	11.15 ± 0.10a	0.10 ± 0.00a	251.47 ± 7.16b	1.23 ± 0.04a
5Se	9.03 ± 0.83b	0.10 ± 0.02a	290.16 ± 17.19a	1.20 ± 0.21a
25Se	7.35 ± 0.72d	0.07 ± 0.01bc	256.21 ± 31.98ab	0.86 ± 0.11b
NaCl	7.24 ± 0.45d	0.06 ± 0.01c	232.48 ± 20.24bc	0.71 ± 0.14bc
NaCl + 1Se	8.60 ± 0.67bc	0.07 ± 0.01b	200.13 ± 18.18cd	0.68 ± 0.09c
NaCl + 5Se	7.99 ± 0.36cd	0.05 ± 0.01c	194.97 ± 27.58d	0.72 ± 0.10bc
NaCl + 25Se	5.17 ± 0.38e	0.04 ± 0.00d	186.40 ± 25.10d	0.47 ± 0.04d

The control plants (CK) were cultured in half-strength Hoagland’s solution. Different treatments were added with or without different concentrations of Se or NaCl. The abbreviations 1 Se, 5 Se and 25 Se indicate 1 μM Na_2_SeO_3_, 5 μM Na_2_SeO_3_ and 25 μM Na_2_SeO_3_, respectively (details are shown in the Materials and Methods). Data are presented as the means ± SDs (n = 4). Different letters within a column represent significant differences (*p* < 0.05).

**Table 4:** Effects of Se on K^+^, Na^+^ and Cl^−^ concentrations in the shoots and roots of non-stressed or salt-stressed maize plants.

Treatments	Shoots				Roots			
	K^+^ (g kg^−1^)	Na^+^ (g kg^−1^)	K^+^/Na^+^	Cl^−^ (g kg^−1^)	K^+^ (g kg^−1^)	Na^+^ (g kg^−1^)	K^+^/Na^+^	Cl^−^ (g kg^−1^)
CK	62.71 ± 2.65a	2.07 ± 0.23b	17.87 ± ab	0.40 ± 0.03b	34.64 ± 3.13a	4.52 ± 0.56c	4.59 + 0.98a	0.19 ± 0.03b
1Se	63.60 ± 3.71a	1.98 ± 0.42b	19.36 ± a	0.41 ± 0.04b	34.43 ± 2.87a	4.68 ± 0.58c	4.35 + 0.40a	0.22 ± 0.03b
5Se	59.69 ± 4.78a	2.17 ± 0.54b	16.76 ± ab	0.43 ± 0.04b	33.03 ± 2.12a	4.84 ± 1.17c	4.20 + 1.20a	0.23 ± 0.04b
25Se	51.47 ± 1.15b	2.11 ± 0.59b	15.13 ± b	0.35 ± 0.04b	28.36 ± 4.75b	4.60 ± 0.84c	3.64 + 0.32a	0.21 ± 0.02b
NaCl	41.44 ± 3.02d	22.57 ± 0.95a	1.08 ± c	3.85 ± 0.18a	13.48 ± 0.97c	33.24 ± 1.72a	0.24 + 0.02b	2.89 ± 0.21a
NaCl + 1Se	52.55 ± 3.59b	22.37 ± 1.59a	1.39 ± c	3.72 ± 0.16a	16.84 ± 1.43c	28.09 ± 0.76b	0.35 + 0.02b	2.73 ± 0.13a
NaCl + 5Se	47.79 ± 5.01bc	21.84 ± 1.21a	1.29 ± c	3.71 ± 0.11a	15.97 ± 1.58c	28.20 ± 1.87b	0.33 + 0.01b	2.78 ± 0.15a
NaCl + 25Se	44.98 ± 2.17cd	22.83 ± 0.48a	1.16 ± c	3.74 ± 0.13a	13.06 ± 1.07c	31.53 ± 1.66a	0.24 + 0.02b	2.88 ± 0.12a

The control plants (CK) were cultured in half-strength Hoagland’s solution. Different treatments were added with or without different concentrations of Se or NaCl. The abbreviations 1 Se, 5 Se and 25 Se indicate 1 μM Na_2_SeO_3_, 5 μM Na_2_SeO_3_ and 25 μM Na_2_SeO_3_, respectively (details are shown in the Materials and Methods). Data are presented as the means ± SDs (n = 3). Different letters within a column represent significant differences (*p* < 0.05).

**Table 5:** Plant treatments in this experiment.

Abbr.	NaCl (mM)	Na_2_SeO_3_ (μM)
CK	0	0
1Se	0	1
5Se	0	5
25Se	0	25
NaCl	100	0
NaCl + 1Se	100	1
NaCl + 5Se	100	5
NaCl + 25Se	100	25

**Table 6:** Sequences of the forward and reverse primers used in qRT-PCR for gene expression analysis in maize plant roots.

Gene name	Forward primer sequence (5′ to 3′)	Reverse primer sequence (5′ to 3′)
MPK5	GATTATCAGTAGCCAAAGTTCAA	ACACCGTCACCAGCTTTTAATC
MPK7	CCAGTAGCCAAAGTTCGGTTC	TACAGACAACACCGAGAAGTACTTA
CPK11	AGAACGAAATCCAGGCTCTAATG	ATTCGCGACATGCTTGTGAC
ZmNHX1	ATGCAGGGTTCCAAGTGAAG	AATATTGCCCCAAGTGCAAG
Actin	GACCTCACCGACCACCTAATG	CTGAACCTTTCTGACCCAATG
